# The Influence of Parental Education on Timing and Type of Union Formation: Changes Over the Life Course and Over Time in the Netherlands

**DOI:** 10.1007/s13524-016-0473-y

**Published:** 2016-06-21

**Authors:** Jarl E. Mooyaart, Aart C. Liefbroer

**Affiliations:** 1 Netherlands Interdisciplinary Demographic Institute, The Hague, The Netherlands; 2 University of Groningen, Groningen, The Netherlands; 3 Department of Epidemiology, University Medical Centre Groningen (UMCG)/University of Groningen, Groningen, The Netherlands; 4 Department of Sociology, Vrije Universiteit Amsterdam, Amsterdam, The Netherlands

**Keywords:** Marriage, Cohabitation, Parental education, Historical change, Life course

## Abstract

Family background shapes young adults’ decisions in their transition to adulthood, and the outcomes of these decisions lay the foundation for their subsequent life course. This study examines the influence of parental education on their children’s union formation. We examine the timing of entry into a first union (a married or a cohabiting union), the choice between marriage and cohabitation, and the timing of first marriage. Data from eight nationally representative surveys conducted in the Netherlands are pooled (*N* = 39,777), with respondents being born between 1930 and 1990, to examine not only the effect of parental education on union formation but also whether this effect changes over birth cohorts, periods, and the life course, and varies by gender. Results from discrete-time hazard analyses show little change in the effect of parental education across cohorts and periods but strong life-course effects. Gender differences in the effect of parental education are relatively small.

## Introduction

Parental educational attainment strongly influences union formation (Axinn and Thornton 1992; Cavanagh 2011; Liefbroer 1991; Mulder et al. 2006; South 2001; Thornton et al. 2008; Uecker and Stokes 2008; Wiik 2009). Young adults with highly educated parents enter their first union (Cavanagh 2011; Mulder et al. 2006; Wiik 2009) and first marriage (Axinn and Thornton 1992; Sassler et al. 2009; South 2001; Uecker and Stokes 2008) at a later age than young adults with relatively low-educated parents. The timing of the first union can have important implications for the subsequent life course. Unions formed at an early age have a higher chance of disruption (Berrington and Diamond 1999; Lyngstad 2006), and union dissolution has been associated with higher risks of unemployment (Covizzi 2008). Furthermore, children born in cohabiting households are more likely to have lived with a single mother compared with those born to married parents (Heuveline et al. 2003). As a result, children of cohabiting parents may end up with fewer resources than children raised within marriage (Manning and Brown 2006; Manning and Lichter 1996). Therefore, examining the influence of parental education on union formation may improve our knowledge about persisting intergenerational social inequality.

In many Western countries, unmarried cohabitation is on the rise, often replacing marriage as the most popular type of first union (Bumpass and Lu 2000; Kiernan 2001). In the Netherlands, the focus of the present study, 83 % of those born between 1970 and 1979 opted for unmarried cohabitation, which is a somewhat lower rate than seen in the Scandinavian countries (86 % in Norway to 94 % in Denmark) but relatively high compared with other Western European countries, such as Germany (74 %) and the United Kingdom (72 %) (Billari and Liefbroer 2010).

The increasing popularity of unmarried cohabitation complicates the analysis of the influence of parental education on union formation. Unmarried cohabitation can serve as both a precursor of marriage and an alternative to it (Berrington and Diamond 2000; Cherlin 2004; Hiekel et al. 2014; Landale and Forste 1991; Wiik 2009). Parents may influence not only the timing of relationship formation but also the choice for the type of first union: that is, married or unmarried cohabitation. Most U.S. research regarding the choice between married and unmarried cohabitation has shown that cohabitation is more common among those from disadvantaged backgrounds (Bumpass and Lu 2000; Kennedy and Bumpass 2008; Manning and Cohen 2015; Seltzer 2004), although some studies have shown no effect of parental education (Lichter et al. 2010; Sassler et al. 2009) or even that cohabitation is more likely among those with higher-educated mothers (Cohen and Manning 2010; Lichter and Qian 2008). Liefbroer (1991) found that in the Netherlands, children with highly educated parents are more likely to opt for unmarried cohabitation. Research from other European countries is scarce and has produced mixed results (Hoem and Kostova 2008; Schröder 2006).

The central focus of this study is the extent to which the effect of parental education on the timing of union formation and on the choice between marriage or unmarried cohabitation as the first union type varies over birth cohorts, periods, the life course, and with gender. Previous research has found that the effect of parental education on timing of relationship formation decreases over the life course and across cohorts (Sassler and Goldscheider 2004; South 2001; Wiik 2009). This study contributes to this literature in four ways. First, it examines the influence of parental education among a broad range of cohorts born between 1930 and 1990. No previous studies have covered such an extensive range of cohorts, allowing us to study whether the influence of parental education attenuated among cohorts that experienced the second demographic transition (SDT) (Lesthaeghe 2010; Lesthaeghe and Surkyn 1988; Lesthaeghe and Van de Kaa 1986). Second, previous research on changes in the influence of parental education over the life course and over time has focused only on the timing of union formation, whereas this study also includes the choice between married or unmarried cohabitation for the first union. Third, this study examines the timing of both the first union and the first marriage. Finally, this study examines not only cohort change but also period change by taking into account national annual changes in economic circumstances.

## Theory

With the rise in unmarried cohabitation, the relationship formation process has become more complex. Before the 1960s, unmarried cohabitation occurred only in rare circumstances; however, today, it is a common form of first union in the Netherlands (Manting 1996) and in many other Western countries (Billari and Liefbroer 2010; Bumpass and Lu 2000). First, we discuss how parental education influences the timing of entry into a first union (either married or unmarried cohabitation) and first marriage. Next, we examine the influence of parental education on the choice between marriage and unmarried cohabitation. Finally, we discuss how these processes may vary by cohort, period, age, and gender.

### Parental Education and the Timing of Union Formation

There are several arguments about why higher parental education leads to postponement of first union and first marriage. Children with highly educated parents may be socialized differently than children with low-educated parents. As theories on the intergenerational transmission of education stipulate, children with educated parents are likely to have higher education and career aspirations (e.g., Dubow et al. 2009; Schoon and Parsons 2002; Sewell and Shah 1968), leading to higher educational attainment and to prolonged enrollment in the educational system (Van Hek et al. 2015; Shavit and Blossfeld 1993). This prolonged enrollment in the educational system leads to the postponement of relationship formation because the educational system serves as a moratorium in which demographic transitions are delayed (Blossfeld and Huinink 1991; Liefbroer and Corijn 1999; Raymore et al. 2001; Thornton et al. 1995).

Given the strong association between education and income (e.g., Ashenfelter and Rouse 2000; Bradbury 2002; Miller et al. 1995), children with highly educated parents are more likely to be raised in a wealthy home environment than children with low-educated parents. Individuals who were raised in a household with high consumption levels may develop the same consumption aspirations for their own household (Easterlin 1980) and may not want to start a household before they are able to afford a similar lifestyle themselves, which will delay their timing of marriage (Axinn and Thornton 1992). In addition, remaining in the parental home longer may be more appealing to children with highly educated parents given that their parental home is likely to provide more nonmaterial (such as a warm psychological climate) and material (such as a larger house and more luxury in the home) resources, making them less inclined to leave the parental home (Axinn and Thornton 1992). Moreover, children with low-educated parents may be more inclined to view entry into a union as a potential route to leave an unsatisfying parental home situation (Clarkberg 1999). Parental resources may also influence the relationship formation for those who already left the parental home. Parents can use their financial resources to influence the timing of the first union by providing better alternatives to early marriage in late adolescence and early adulthood (Manting 1996; Sassler and Goldscheider 2004; Waite et al. 1986). Therefore, we expected the following:

- *Hypothesis 1*: The higher the parents’ level of educational attainment, the higher the age of entry into first union and first marriage of their children.

Until now, we assumed that parental education affects the timing of cohabiting and marriage in comparable ways. However, given that marriage is less easily reversible and more consequential than cohabitation, perhaps parents are more involved with their children’s marriage timing than their timing of cohabitation (Wiik 2009). In addition, given the high costs of marriage, parental financial support may be more important for the decision to marry. Both arguments lead one to expect that the influence of parental education on the timing of marriage is somewhat stronger on marriage than on cohabitation. On the other hand, given that in the Netherlands cohabitation often precedes marriage (Statistics Netherlands 2006), one could argue that the influence of parents on marriage timing may be weaker because by the time of first marriage, children will be less dependent on their parents. Wiik (2009) did not find differences in the effect of parental education on whether the first union is a cohabiting or marital relationship. Thus, we will not formulate a specific hypothesis on this issue but explore the issue in our empirical analysis.

### Parental Education and the Choice Between Marriage and Cohabitation

Parents’ educational attainment may also influence whether their children opt for marriage or unmarried cohabitation when they first enter a union. The literature is divided about whether children with an advantaged or a disadvantaged background opt for cohabitation. One popular idea is that cohabitation is a type of “poor man’s marriage,” in which young adult men and women engage who do not have the financial resources to enter marriage (yet) (Hiekel et al. 2014; Perelli-Harris et al. 2010). Young adults with low-educated parents are likely to have fewer resources than their peers with highly educated parents. Thus, lower parental education would result in a higher propensity to opt for unmarried cohabitation rather than direct marriage. Research from the United States (Bumpass and Lu 2000; Kennedy and Bumpass 2008; Lichter et al. 2006; Manning and Cohen 2015; Seltzer 2004), and Bulgaria (Hoem and Kostova 2008) supports this idea. In contrast, the SDT theory claims that the choice for unmarried cohabitation is based on a cultural preference rather than structural constraint, with those who are more individualistic and less traditional being more likely to opt for this relationship form (Lesthaeghe 2010). Higher education has been associated with having less-orthodox family and marital values, including less disapproval of unmarried cohabitation (De Valk and Liefbroer 2007; Liefbroer and Billari 2010; Van der Valk et al. 2008). Thus, highly educated parents are likely to socialize their children with these more liberal values, implying that their children are more likely to opt for unmarried cohabitation. In the Netherlands (Liefbroer 1991) and Italy (Schröder 2006), children with highly educated parents are more likely to opt for unmarried cohabitation for their first union. Furthermore, although much research has indicated that lower education is associated with a higher propensity for unmarried cohabitation, some research in the United States has suggested that those with highly educated mothers are more prone to single-instance and serial cohabiting (Cohen and Manning 2010; Lichter and Qian 2008).

In the Netherlands, low parental education may be less strongly associated with unmarried cohabitation than in other countries for two reasons. First, the Netherlands is a country with relatively little poverty and high welfare expenditure (Caminada et al. 2012; Peichl et al. 2010). Thus, even young adults with limited parental resources are likely to have the means to marry. Second, in the Netherlands, teenage births and births to single mothers are much less common than in the United States and many other European countries (Ellwood and Jencks 2004; Perelli‐Harris et al. 2010; Robson and Berthoud 2003; Santelli and Melnikas 2010). Thus, the pool of young adults from a low class background that is most likely to opt for cohabitation is simply smaller in the Netherlands than in other countries. Therefore, we expect the following:

- *Hypothesis 2*: The higher the parents’ level of educational attainment, the more likely that their children will enter their first union by unmarried cohabitation rather than by direct marriage.

### Variability in the Influence of Parental Education

#### Cohort Changes

In the twentieth century, both cultural and structural changes occurred in the Netherlands that likely decreased the influence of parental education on their children’s union formation decisions. First, SDT theory claims that around the 1960s, a cultural shift occurred in which values of solidarity and social group adherence lost their prominent position to values of autonomy and self-realization (Lesthaeghe 2010; Lesthaeghe and Van de Kaa 1986). Parents reevaluated their role in socialization, placing more emphasis on stimulation and autonomy rather than on discipline (Sieben and De Graaf 2003; Van Poppel et al. 2008). Moreover, parents became less able and willing to exert social pressure on their children (Kalmijn 1998). Although unmarried cohabitation is still less popular among religious people (Jansen 2002), the Netherlands became more secularized in the 1960s (Becker and De Wit, 2000), increasing the acceptance of unmarried cohabitation among all social strata. These cultural shifts are likely to have decreased the role of parents in their children’s decisions regarding living arrangements and parenthood.

Structural societal changes may also account for the potential decline in the influence that parents have over their children’s relationship formation behavior. Educational expansion and the rise of the welfare state increased the ability of young adults to provide for themselves without requiring the use of parental resources. Furthermore, the association between parental education and children’s education may have decreased as a result of more equal access to education for children with highly educated and low-educated parents. There is indeed some evidence that educational attainment has become more meritocratic in the Netherlands (van Hek et al. 2015). If children with low-educated parents become increasingly enrolled in education, they will also postpone union formation. Therefore, we expect the following:

- *Hypothesis 3*: The effect of parent’s level of educational attainment on children’s union formation decisions decreases across cohorts.

#### Period Change

Although cultural and structural changes may have led to a decline across cohorts in the influence of parental education on union formation behavior, there may have been some period fluctuations in the effect of parents linked to business cycle effects. Although overall prosperity has increased over the last half-century, the Netherlands has been hit by several economic crises. The crisis in the 1970s and early 1980s was caused by the global oil crisis, and the most recent one starting in 2008 was caused by the global credit crisis. The economic consequences of these crises included an increase in (youth) unemployment, stagnation, a decrease in wages, and increased difficulty in obtaining a mortgage (Bagheloe-Datadin 2013). During the last crisis, the timing of marriage and parenthood has been postponed (de Beer 2012). In times of financial hardship, young adults may have to rely more on their parental resources. As a result, the parents may increase their influence on the union formation decisions of their children: for instance, by supporting them in buying a house (Mulder and Smits 1999). The better educated parents are, the more resources they are likely to have, which may especially make a difference during times of economic hardship. Thus, the influence of parental education is likely to increase in times of economic crisis and decrease in time of economic prosperity, leading to the following hypothesis:

- *Hypothesis 4*: The better the economic circumstances are, the smaller the effect of parents’ level of educational attainment is on their children’s union formation decisions.

#### Life Course Changes

The influence of parents on their children is likely to change during their children’s life course. Although highly educated parents may try to prevent early union formation, they may stimulate union formation later in young adulthood by providing the necessary means for marriage (Manting 1996; Sassler and Goldscheider 2004; Waite and Spitze 1981). However, several arguments have suggested that the influence of parents on their children decreases with age. Young adults reexamine their worldviews and increasingly start adopting their own beliefs based on independent reflection (Arnett 2000). Furthermore, on their path to adulthood, the importance of young adults’ own life experiences and preferences increases relative to features of family background (Hogan and Astone 1986; South 2001). Life events, such as leaving the parental home and obtaining a full-time job, may alter the relationship between parents and children. When children leave home, geographical distance decreases the influence that parents have on their children. Bucx et al. (2012), for instance, showed that children who live independently receive less counsel or personal advice from their parents. Individuals will gain financial independence when they enter full-time employment, enabling them to rely on their own resources and to be less reliant on parental resources. Furthermore, considering first marriage, those who are already cohabiting are likely to be less influenced by their parents because they may (at least partly) rely on the resources of their partner. All these arguments suggest that the influence of parental characteristics, such as parental education, is likely to decrease across young adulthood. This leads to the following hypothesis:

- *Hypothesis 5*: The effect of parent’s level of educational attainment on their children’s union formation decisions decreases over the life course.

#### Gender Differences

Women enter unions earlier than men (e.g., Waite et al. 1986; Uecker and Stokes 2008; Winkler-Dworak and Toulemon 2007). However, few studies have considered whether the influence of parental education has a gender gradient (Axinn and Thornton 1992; Michael and Tuma 1985; Wiik 2009). Highly educated parents may place more pressure on daughters to postpone family formation and focus on their career, knowing that these are more difficult to combine for women given that they are likely to have a larger share in childcare responsibilities than men (Barber 2000; Wiik 2009). However, Wiik (2009) did not find any evidence that this is the case in Norway for those who entered a union between 1970 and 2002. In the United States, Michael and Tuma (1985) found stronger effects for women than for men, but Axinn and Thornton (1992) did not find substantial gender differences.

Mothers and fathers may also differ in their influence on their sons and daughters. Fathers are found to be more involved with sons than with daughters (Harris et al. 1998; Starrels 1994), but for mothers, it is the other way around (Dornbusch 1989; Steinberg 1987). If so, the effect of father’s education may be stronger on sons’ union formation decisions than on those of daughters, and the opposite may be true for mother’s education. However, Russell and Saebel (1997) argued that it is not clear how strong the differences are between the four possible parent–child dyads (mother–daughter, mother–son, father–daughter, father–son). In sum, there is little direct evidence that the influence of parental educational attainment on the union formation process differs by gender of the child or of the parent. Therefore, we will not formulate a hypothesis on gender differences but rather empirically explore whether gender differences are observed.

## Data and Methods

### Data

Data from eight Dutch surveys containing retrospective partner histories were pooled and include four waves (1993, 1998, 2003, 2008) of the Dutch Fertility and Family survey (Onderzoek Gezinsvorming (OG)) (Statistics Netherlands 2008), two waves of the Family Survey Dutch Population (Familie-enquête (FE)) of the year 2003 (De Graaf et al. 2003) and 2009 (Kraaykamp et al. 2009), the Living Arrangements and Social Networks of Older Adults survey in 1992 (NESTOR) (Knipscheer et al. n.d), the ESR telepanel of 1992 (ESR/STP 1992), and selected respondents born in 1930 or later. All surveys are based on probability sampling techniques to assure that they are nationally representative. Nonresponse rates vary considerably between the surveys (see Table 1). To cover for nonresponse, weights were included in the analysis. For all surveys, weights were based on at least the following characteristics: sex, age, marital status, and region or level of urbanization. The age of respondents varies between the data sets. In NESTOR, respondents from the age of 54 were interviewed; in the other data sets, individuals aged 18 and older were included. OG 1993 included only those individuals aged 18–42, but in the other waves of the OG surveys, the upper age limit was 52 in OG 1998 and 62 in OG 2003 and OG 2008. In the other surveys, the maximum age lies at least at age 70. In general, women are slightly overrepresented, with a maximum of 55 % women in FE 2003. The total number of observations in our study is 39,777.

**Table 1:** An overview of the surveys used in this study

	Nonresponse Rate (%)	Age Range	Percentage of Women
NESTOR 1992	38	54–89	51
ESR Telepanel 1993	43	18–89	48
OG 1993^a^	50	18–42	55
OG1998	27	18–52	54
OG 2003	43	18–62	52
OG 2008	40	18–62	51
FE 2003	47	18–70	55
FE 2009	49	18–90	51

^a^Survey description states a nonresponse of at least 50 %.

Missing values on respondent’s, mother’s, and father’s level of educational attainment were treated by using multiple imputation methods. We opted for predictive mean matching (PMM) because of the skewed distribution of mother’s and father’s education. Another advantage of using PMM is that it imputes only those values already in the data rather than out-of-range values (such as negative values). Values for parents’ and respondent’s education were predicted using gender, birth year, the union formation outcome variable, and the Nelson-Aalen estimator.^1^ The standard PMM matching technique imputes a value from the observation that has the nearest *z* value. One can, however, increase the number of potential donors by selecting a random pick from a *k* number of nearest donors. In our analysis, a *k* value of 10 is used as suggested by Morris et al. (2014). The data are imputed 10 times, and the results from the imputed data sets are combined using Rubin’s (1987) rules.

### Measures

In all surveys, respondents were asked to report the start and end dates (in years and months) of all their cohabiting (married or unmarried) relationships that lasted at least three months. Based on this information, the three dependent variables (timing of entry into a first union, timing of entry into first marriage, and whether the first union was entered by marriage or by unmarried cohabitation) were constructed.

The main independent variables are father’s and mother’s level of educational attainment. Because level of education was coded slightly different in each survey, a strategy had to be adopted to recode these variables into a uniform measure of education. Some OG surveys used broad categories with scores ranging from 1 (primary education or less) to 5 (university), while the FE surveys and the ESR telepanel had (respectively) 10 and 8 educational level categories. In NESTOR, the education variables indicated the number of years of education. We chose to create a continuous measure for education using the International Standard Level of Education (ISLED) (Schröder and Ganzeboom 2013). The ISLED is a continuous measure of education that allows comparison across surveys and across countries. For all these categories, ISLED scores were matched (see appendix, Table 8). When more than one ISLED score could be matched to a category, the average of all the different ISLED scores that were covered by a category was taken.

For respondents themselves, we also use information on their highest educational attainment. However, because using highest education as a time-constant variable could lead to estimation bias (Hoem and Kreyenfeld 2006), we created a time-varying incremental ISLED score in which respondents have a lower ISLED at younger ages based on where they are in the Dutch educational system at that age, and only reach their reported highest level of education at the youngest age at which this would be possible, given the structure of the Dutch educational system.^2^

**Fig. 1:**
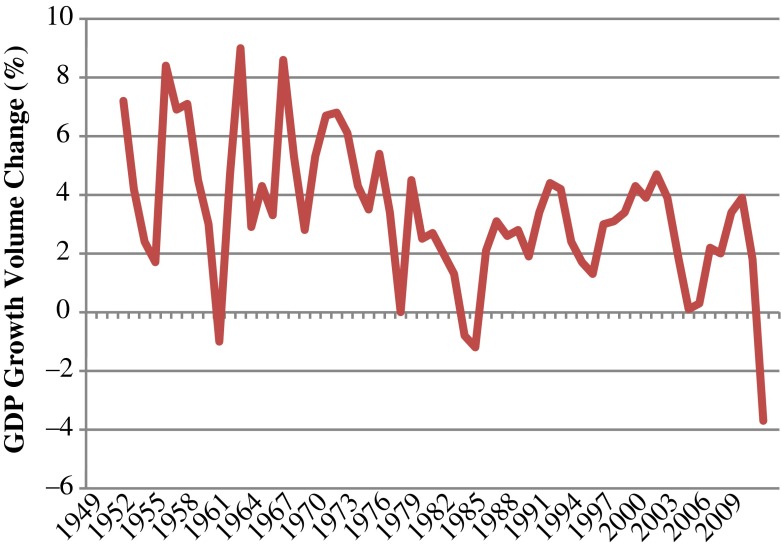
Development of GDP growth volume change from 1949 to 2009

The variables that are interacted with father’s and mother’s education are *age*, *cohort*, *economic growth*, *female*; and for the timing of first marriage, also the variable *cohabitation*. The *age* variable is constructed as the number of years since age 15 until one experiences a transition.^3^ To examine whether there have been changes over time, a continuous *cohort* variable is included, using the birth year of the respondent. *Economic growth* is measured by GDP volume change (percentage). For GDP, yearly information from 1949 until 2009 is available from Statistics Netherlands (2012). Figure 1 shows the trend in economic growth. In our models, the economic growth measure is lagged by one year. The *female* variable is coded 0 for males and 1 for females. In the analysis of timing of first marriage, *cohabitation* is a time-varying dichotomous variable indicating whether someone at a certain age is in a cohabiting relationship.

Finally, some controls are included in the analysis. First, the religious affiliation of both mother and father is incorporated, categorized as 0 = no religion (reference category), 1 = Catholic, 2 = Protestant, 3 = other religion, or 4 = missing. Second, a dummy variable indicating whether the respondent experienced a parental divorce before age 18 is included. Finally, we control for possible survey differences by including a series of dummy variables for each of the surveys (OG 1998 = reference category). Descriptive information on all dependent and independent variables are shown in Table 2.

**Table 2:** Descriptive statistics of independent variables^a^

Variables	Mean (SD)	Range	*N*
Cohort	1961.73 (11.64)	1930–1990	39,777
Female (ref. = male)	0.53	0/1	39,777
NESTOR	0.02	0/1	39,777
ESR Telepanel	0.04	0/1	39,777
OG 1993	0.21	0/1	39,777
OG 1998	0.26	0/1	39,777
OG 2003	0.20	0/1	39,777
OG 2008	0.20	0/1	39,777
FE 2003	0.03	0/1	39,777
FE 2009	0.05	0/1	39,777
Father No Religion	0.21	0/1	39,777
Father Catholic	0.38	0/1	39,777
Father Protestant	0.26	0/1	39,777
Father Other Religion	0.07	0/1	39,777
Father Missing Religion	0.09	0/1	39,777
Mother No Religion	0.18	0/1	39,777
Mother Catholic	0.40	0/1	39,777
Mother Protestant	0.28	0/1	39,777
Mother Other Religion	0.07	0/1	39,777
Mother Missing Religion	0.07	0/1	39,777
Divorced Parents <18	0.05	0/1	39,777
Father’s Education	42.69 (22.58)	16.55–92.63	34,368
Mother’s Education	35.17 (17.58)	16.55–92.63	35,592
Respondent’s Education	58.31 (19.13)	16.55–94.62	39,334

^a^More detailed information on the age of entry into first union and first marriage is provided in Table 3.

### Analytical Strategy

The data are organized in a person-period file (Allison 1984), with separate records for each month that an individual was at risk, starting from age 15. If respondents do not experience entry into a union or entry into marriage, they are censored when they reach age 40 or at the time of interview, whichever comes first. Discrete-time (logistic regression) hazard models are estimated for entry into first union and entry in first marriage. A multinomial logistic regression model is estimated for the choice between married and unmarried cohabitation.

For all analyses, three models are presented. Model A is the base model and includes only the main independent variables and controls but not respondents’ own educational attainment. For age and cohort, quadratic and cubic terms are included.^4^ Age is cubed because union rates decrease at older ages. Cohort is cubed because the changes in union rates may not be linear. In fact, they show a dramatic increase around the 1960s and then more or less stabilize thereafter. The model also controls for differences in men’s and women’s age patterns and cohort changes of union formation by interacting female with age, age^2^, age^3^, cohort, cohort^2^, and cohort^3^. In Model B, respondents’ own level of education is included to examine the extent to which the influence of parental education is mediated by respondents’ own educational attainment. Model C includes interactions of parental education with cohort, economic growth, age, and female. In the analysis of entry into first marriage, parental education is also interacted with unmarried cohabitation to examine whether this life-course event changes the influence that parental education has on marriage timing of their children.

## Results

Table 3 presents the median age of entry into first union and first marriage as well as the percentage of first unions that started as an unmarried cohabitation, by gender, cohort, and parents’ level of education. Educational level is split into those with low (i.e., at most lower vocational education (ISLED ≤ 29.34)), middle (i.e., those who have an educational level somewhere in between (ISLED > 29.34 and ISLED < 77.92)), and high (i.e., those with at least some finished tertiary education (ISLED ≥ 77.92)). Two cohorts are distinguished: those born before 1960 and those born since then. Table 3 shows that in general, the median age at first union has remained fairly stable across cohorts: that is, for women and men at approximately 22 and 25 years, respectively. However, the median age at first marriage is much higher for men and women born since 1960 compared with those born before 1960. One-half of the women and men born before 1960 had already married by ages 23 and 26, respectively, whereas the median ages for men and women born after 1960 increased to approximately 27 and 31, respectively. Finally, men and women born after 1960 were much more likely to opt for unmarried cohabitation as their first union compared with those born before 1960. About one-third of those born before 1960 chose unmarried cohabitation, whereas more than two-thirds of those born after 1960 did so. In both cohorts, men are slightly more likely than women to enter a cohabiting union. These gender differences arise because men generally are somewhat older (and thus are a member of an earlier birth cohort) at entry into a first union than their female partner. As a result, a shift toward unmarried cohabitation will occur a few birth cohorts earlier among men than among women.

**Table 3:** Median age of entry into first union and first marriage, and the percentage of unmarried cohabitation by gender, cohort, and father’s and mother’s education

	Low-Education Father	Middle-Education Father	High-Education Father	Low-Education Mother	Middle-Education Mother	High-Education Mother	Total
Median Age at First Union							
Women 1930–1960	22.1	22.3	23.3	22.1	22.5	23.7	22.2
Women 1960–1990	22.2	22.7	23.6	22.3	22.8	23.9	22.6
Men 1930–1960	24.5	24.5	24.9	24.4	24.9	24.8	24.5
Men 1960–1990	25.0	25.2	25.5	25.0	25.3	25.7	25.3
Median Age at First Marriage							
Women 1930–1960	22.4	23.3	25.5	22.5	24.1	26.3	22.8
Women 1960–1990	25.9	27.4	30.2	26.1	28.3	30.9	27.2
Men 1930–1960	25.1	26.0	27.6	25.2	27.3	27.8	25.5
Men 1960–1990	29.8	30.9	32.4	30.0	31.6	33.9	31.0
% Cohabitation as First Union							
Women 1930–1960	20.3	35.2	51.2	22.0	45.5	56.7	27.1
Women 1960–1990	66.3	78.4	84.9	69.1	80.1	90.1	73.3
Men 1930–1960	25.8	45.2	56.2	28.7	52.3	61.4	34.1
Men 1960–1990	74.9	81.2	86.1	75.8	85.1	88.8	78.8

Table 3 also shows differences in union formation by level of parental education. For both men and women, the more highly educated the mother and father are, the higher the median age of entry into first union and first marriage is; the only exception is that men born before 1960 who have a highly educated mother have a slightly lower median age of entry into first union than men who have a middle-level-educated mother. The median age differences between parental educational groups are larger for first marriage than for first union. For women, there is only about a one-year difference in median age of first union between those with high- and low-educated mothers and fathers; for men, this difference is smaller. For first marriage, these differences range from about 2.5 to 4.5 years, again with somewhat smaller differences for men than for women. There appears to be little cohort change in educational background differences in entry into a union and marriage. The increase in the median age at first marriage in the youngest cohort is observed among all parental education groups, which implies that relative differences remain about the same. In general, median age differences appear to be slightly larger for mother’s than for father’s education.

The percentage of men and women who enter their first union by unmarried cohabitation also varies considerably by parents’ education. In the 1930–1960 cohort, approximately one-quarter of those with a low-educated parent opt for unmarried cohabitation, and approximately one-half of those with a highly educated parent do so. In the 1960–1990 cohort, the proportion of individuals with a low-educated parent who opt for unmarried cohabitation as a first union rises to about two-thirds for women and about three-quarters for men; for those with a highly educated parent, it increases to more than 80 % for both men and women. For both men and women, the relative differences between those with low-educated and highly educated fathers and/or mothers decrease over the two cohorts. In sum, these descriptive results suggest that although the entry into first union and first marriage has been postponed among all groups, a parental educational gradient remains. The same applies to the choice between marriage and cohabitation.

In Table 4, the effects of parental educational attainment—and its relevant interactions—on the rate of entry into first union and first marriage and on the choice for cohabitation versus marriage are presented. In Table 4, Models A, entry into a first union and entry into a first marriage show significant effects for both father’s and mother’s education. Every additional ISLED point of father’s and mother’s education decreases the rate of entering a first union by, respectively, 0.3 % and 0.5 %. For first marriage, these figures are somewhat larger (0.7 % and 0.8 % per ISLED point, respectively). This confirms Hypothesis 1, in that higher parental education is associated with a delay of both first union and first marriage. Regarding the choice between cohabitation and marriage, Model A shows that an increase of one ISLED point for father’s and mother’s education is associated with, respectively, a 1.0% and a 1.1 % increase in the odds of choosing unmarried cohabitation rather than marriage at entry into a first union. These results confirm Hypothesis 2: that is, children with highly educated parents are more likely to opt for unmarried cohabitation. In all three analyses, Models B of Table 4 show that respondents’ own level of education has the same type of effects as parental education, but also that the effects of father’s and mother’s level of education are only slightly reduced if respondent’s own level of education is included.

**Table 4:** Results of discrete-time and multinomial logistic regression on the influence of father’s and mother’s education on union formation

	First Union			First Marriage			Cohabitation vs. Marriage		
	Model A	Model B	Model C	Model A	Model B	Model C	Model A	Model B	Model C
	*b* (SE)	*b* (SE)	*b* (SE)	*b* (SE)	*b* (SE)	*b* (SE)	*b* (SE)	*b* (SE)	*b* (SE)
Father’s Education	–0.0032**	–0.0023**	–0.0002	–0.0066**	–0.0051**	–0.0084**	0.0098**	0.0076**	0.0095**
	(0.0003)	(0.0004)	(0.0008)	(0.0004)	(0.0005)	(0.0011)	(0.0007)	(0.0007)	(0.0016)
Mother’s Education	–0.0049**	–0.0042**	–0.0035**	–0.0077**	–0.0065**	–0.0146**	0.0111**	0.0095**	0.0109**
	(0.0005)	(0.0005)	(0.0011)	(0.0006)	(0.0006)	(0.0016)	(0.0010)	(0.0010)	(0.0023)
Respondent’s Education		–0.0048**	–0.0050**		–0.0072**	–0.0073**		0.0101**	0.0099**
		(0.0004)	(0.0004)		(0.0004)	(0.0004)		(0.0008)	(0.0008)
Interactions									
Father’s education × Cohort			–0.0000			0.0000			0.0002*
			(0.0000)			(0.0000)			(0.0001)
Father’s education × Economic growth			–0.0002			0.0006**			0.0002
			(0.0002)			(0.0002)			(0.0004)
Father’s education × Age			0.0005**			0.0007**			–0.0003
			(0.0001)			(0.0001)			(0.0002)
Father’s education × Female			–0.0028**			–0.0007			–0.0000
			(0.0007)			(0.0009)			(0.0016)
Father’s education × Cohabitation						0.0045**			
						(0.0009)			
Mother’s education × Cohort			0.0000			–0.0002**			–0.0000
			(0.0001)			(0.0001)			(0.0001)
Mother’s education × Economic growth			–0.0003			0.0000			0.0004
			(0.0003)			(0.0003)			(0.0006)
Mother’s education × Age			0.0005**			0.0010**			–0.0009**
			(0.0001)			(0.0001)			(0.0003)
Mother’s education × Female			0.0005			0.0031*			0.0006
			(0.0010)			(0.0013)			(0.0023)
Mother’s education × Cohabitation						0.0102**			
						(0.0012)			
Chi-Squared Test^a^	391.85**	163.94**	166.93**	748.71**	302.37**	514.04**	938.25**	356.93**	320.60**
df	2	1	8	2	1	10	4	2	16

*Note:* For all controls included see Tables 5–7 in the appendix.

^a^Wald test: Model A, comparing this model with a model with only controls; Model B comparing with Model A; and Model C comparing with Model B. df = degrees of freedom (indicates the number of additional variables in the respective model compared with the previous). For the multinomial regression, this number is doubled because there is a Cohabitation versus Marriage model and a Single versus Marriage model (see Appendix).

*p < .05; **p < .01

In Models C of Table 4, interactions between father’s and mother’s education and age, cohort, economic growth, and female are added to the model in order to test variations in the effect of parental education. First, we examine interactions between parental education and cohort to test Hypothesis 3: that is, the effect of parental education decreases over cohorts. The results offer little support for this hypothesis. The only significant effect in the expected direction is observed in the multinomial model, where the positive effect of father’s education on the choice for unmarried cohabitation decreases across cohorts. Contrary to expectations, we also observe a statistically significant negative interaction between mother’s education and cohort in the analysis of first marriage, indicating that the delaying effect of mother’s education on the timing of first marriage has increased rather than decreased across cohorts.

Hypothesis 4 states that with better economic circumstances, the effect of parental education decreases. To test this hypothesis, we include interactions between father’s and mother’s level of educational attainment and the level of economic growth in Table 4, Models C. Only one of these interactions is statistically significant: the delaying effect of father’s education on the timing of the first marriage becomes smaller when economic circumstances improve. Thus, we find only weak support for Hypothesis 4.

The fifth hypothesis states that the effect of parental level of educational attainment decreases over the life course. To test this hypothesis, we include interactions between father’s and mother’s level of education and the child’s age in Table 4, Models C. These interactions are positive and statistically significant for entry into first union and entry into first marriage, implying that the delaying effect of father’s and mother’s education on entry into a first union and entry into first marriage attenuates as their child grows older. Regarding the choice between married and unmarried cohabitation, we find a negative and statistically significant effect for the interaction between mother’s education and age, indicating that the increased likelihood to choose unmarried cohabitation decreases as children age. The interaction between age and father’s education is also negative, but it is not significant. In addition, by including an interaction between father’s and mother’s education and cohabitation, we test whether the effect of parental education on the timing of entry of marriage is weaker for those young adults who are already cohabiting. This interaction is positive and statistically significant, indicating that the delaying effect of father’s and mother’s education is weaker after the child has entered a cohabiting relationship.^5^ Thus, overall, we find strong support for Hypothesis 5.

In the Theory section, we discussed the possibility of gender differences in the effect of parental education. Therefore, we test whether the effects of mother’s and father’s education differ and interacted father’s and mother’s education with gender. Regarding the difference between father’s and mother’s education, additional Wald tests (not shown in table) reveal that the effect of mother’s education is stronger than father’s education for the timing of the first union (χ^2^(1) = 5.42, *p* < .05) and for the choice between cohabitation and marriage (χ^2^(1) = 10.58, *p* < .01) but not for the timing of first marriage (χ^2^(1) = 1.53, *p* > .10). The interactions with gender reveal that for first union, there is an effect only of father’s education for women; for first marriage, the effect of mother’s education is stronger for men than for women. Regarding the choice between marriage and cohabitation, no significant differences exist in the strength of father’s or mother’s education between men and women.

Finally, examining the effects of some controls (presented in Tables 5, 6, and 7 in the appendix) shows that those with religious parents are more likely to choose marriage rather than cohabitation as their first union and to enter marriage earlier. Having experienced a parental divorce before age 18 accelerates entry into first union, leads to a postponement of marriage, and increases the likelihood of choosing unmarried cohabitation as the first union. Finally, in bad economic times, people are more likely to postpone union formation and to opt for cohabitation rather than marriage as their first union type.

## Summary and Discussion

The aim of this study was to examine how parental educational attainment influences the union formation process, and to what extent this influence varies by cohort, period, life course, and gender. Because of the rise in unmarried cohabitation, we examined the influence of parental education on three aspects of the union formation process: (1) the timing of the start of the first union (irrespective of whether this was an unmarried cohabitation or a marriage), (2) the timing of first marriage, and (3) whether the first union was entered as an unmarried cohabitation or a marriage. The study was conducted in the Netherlands, which can be considered a country with relatively high levels of unmarried cohabitation.

In line with Hypothesis 1, individuals with highly educated parents postpone entry into first union and first marriage compared with those with lower-educated parents. This finding is consistent with previous research on the timing of first unions (Cavanagh 2011; Mulder et al. 2006; Wiik 2009) and of first marriage (Axinn and Thornton 1992; South 2001; Uecker and Stokes 2008). Also in line with previous studies, the effect of parental education is only partially mediated by children’s own educational attainment (Cavanagh 2011; Wiik 2009), implying that the influence of educated parents is not just a result of the intergenerational transmission of education. Although not hypothesized, the effects of parental education appear stronger for first marriage than for first union. Given that the consequences of the decision to marry are often somewhat greater than those of the decision to cohabit, perhaps parents put more effort in trying to influence the decision to marry.

Higher parental education is also associated with increased odds of choosing unmarried cohabitation rather than marriage as a first union, which is in line with Hypothesis 2. This confirms previous research in the Netherlands (Liefbroer 1991) but runs counter to research in the United States and Eastern Europe, where lower education is associated with the choice of unmarried cohabitation as a first union (Bumpass and Lu 2000; Hoem and Kostova 2008; Kennedy and Bumpass 2008; Lichter et al. 2006; Manning and Cohen 2015; Perelli-Harris and Gerber 2011; Seltzer 2004). In the Netherlands, as well as in some other Western European countries, opting for cohabitation as a first union may be mainly an expression of individualistic preferences rather than a result of economic circumstances (Hiekel et al. 2014).

This study used a long historical time range, including birth cohorts from 1930 to 1990, meaning that individuals entering a union before the presumed start of the SDT were included. It was expected, as stated in Hypothesis 3, that the influence of parental education would decrease across birth cohorts. However, the results of this study suggest that the influence has remained stable with only two exceptions. First, in line with expectations, the effect of father’s education on the choice between cohabitation and marriage decreases across birth cohorts. Second, and contrary to our expectations, this study finds that the delaying effect of mother’s education increases across cohorts. This result is difficult to explain, but it may be related to the fact that relatively few mothers among older cohorts in our study had reached a high level of education. As a result, mother’s educational attainment might have become a more important distinguishing feature among younger cohorts in our study. Not finding a decreasing effect of parental education over time contrasts with results from previous studies using data from the United States and Norway (Sassler and Goldscheider 2004; South 2001; Wiik 2009), which have not found a decreasing effect of parental education over time. However, the empirical evidence is not conclusive. Studies have found this decrease over time either for entry into first marriage (Sassler and Goldscheider 2004; South 2001) or first union (Wiik 2009) only in two national contexts. Furthermore, Wiik (2009) found that the influence of mother and not of the father decreases, whereas South (2001) did not find mother’s educational level to be significant in all models.

Not only do we find little change in the effect of parental education across cohorts, but also period-related changes in the economy do not appear to alter the effect of parental education much. According to Hypothesis 4, the better the economic circumstances are, the weaker the influence parental education would be. However, only the effect of father’s education on first marriage is found to be significantly weaker the better the economic circumstances, providing very limited support for Hypothesis 4. Thus, neither cultural nor economic changes in the second half of the last century appear to have changed the effect of parental education on union formation behavior. This finding is in contrast with the SDT theory, according to which the process of individualization would ultimately diminish the role of parental education on relationship formation. The absence of change in the effect of parental education over time may result for two reasons. First, although normative influence may have decreased, parents may still use their financial resources to avoid early marriage or cohabitation for their children, even in times of an economic crisis. Second, rather than a decline in adherence to social norms, new norms may have emerged that differ between social classes. Liefbroer and Billari (2010) indicated that the higher educated have developed a new set of norms that include preferences for spending a period living independently, a period of unmarried cohabitation, and the postponement of childbearing. Moreover, childbearing within cohabitation has become increasingly common among the lower-educated in Europe (Perelli‐Harris et al. 2010). Thus, although norms and behaviors change, differences between individuals with high- or low-education parents may remain.

Regarding changes over the life course, as expected in Hypothesis 5, the effect of education of the parents on timing of the first union and first marriage decreases with age, which is in line with previous research (South 2001; Wiik 2009). Furthermore, the influence of mother’s education on choice for cohabitation and marriage also decreases with age, although we do not observe the same for father’s education. In addition, unmarried cohabitation decreases the effect of parental education on the timing of first marriage, indicating that life-course events—such as the start of an unmarried cohabiting relationship—decrease the influence of parents on their children’s marriage timing. Thus, strong evidence exists for the importance of parental education mainly in the early phases of young adulthood.

The results on gender differences generally show that mother’s level of education matters more than father’s level of education, at least with regard to the timing of first union and the choice between marriage and cohabitation. One reason could be that Dutch mothers invest more in childrearing than do fathers. If so, mother’s level of education could also be expected to more strongly influence other decisions in young adulthood—for instance, in the employment domain. Alternatively, perhaps this stronger effect of mothers is mainly limited to family formation. Classical thinking on parental socialization suggests that mothers are more influential in the family domain, whereas fathers are more influential in the employment domain (Aldous and Hill 1965). This reasoning could particularly apply to a country like the Netherlands that has long been characterized by a fairly traditional division of labor. The effects of parental education on sons and daughters are generally comparable, with only two exceptions. Father’s educational attainment does not influence their son’s union formation timing at all, and mother’s educational attainment is particularly important for entry into marriage among sons. Although it is difficult to suggest a convincing explanation for these exceptions, the general storyline is that both sons and daughters are influenced by their parents’ educational attainment.

This study has a number of limitations. First, we were not able to distinguish to what extent the influence of parental education can be attributed to financial resources or socialization because most surveys did not contain information on family income, occupational status of the parents, or both. Second, we used a national estimator for economic conditions for young adults, whereas a measure focusing specifically on the economic conditions of young adults would have been preferable. For instance, information on youth unemployment would have been a better indicator. However, there was no information on youth unemployment earlier than the 1970s. Third, our measure of respondent’s own education was constructed as a time-varying education variable, based on the final educational level, whereas the inclusion of a school enrollment variable would have been preferable. However, no data on the timing of actual school enrollment was available. Those enrolled in school are likely to postpone both cohabitation and marriage (Blossfeld and Huinink 1991; Raymore et al. 2001; Thornton et al. 1995). Although not central to our research concerns, it would have been interesting to show how the structural effect of enrollment and the more cultural effect captured by children’s own attained educational level influence both timing and choice of the relationship formation. Finally, this study used retrospective union history data, which implies that results have to be interpreted with some caution given that respondents who entered a first union very long ago might be more likely to underreport such unions—particularly if the union only lasted for a short period of time—than respondents who entered their first union rather recently (Hayford and Morgan 2008). However, as Hayford and Morgan (2008) recommended, we did control for survey differences in our analyses.

In summary, the key findings are that the influence of parental education on their children’s union formation decisions is sizable and has hardly changed over time but becomes weaker as children grow older. Future research on life-course-related changes in the effect of parental education should aim to disentangle whether the influence of family characteristics changes because of a gradual psychological maturation process or the experience of demographic transitions. Furthermore, future research could also examine life-course changes in the association between parental background and other demographic transitions, such as parenthood and divorce. Finally, internationally comparative research is important in order to explain differences between countries in the influence of parental education on union formation behavior. In countries with higher welfare expenditure, individuals may have less difficulty affording marriage, which may make parental resources less important. Cultural differences could be important as well. For instance, in the United States, 74 % of marriages are church weddings (Cherlin 2004) compared with 58 % in the Netherlands (Kalmijn 2004). Because church weddings are, on average, more costly than civil marriages (Kalmijn 2004), parental financial resources may be more important in the timing and occurrence of marriage in the United States than in the Netherlands. Expanding research in these directions will provide a clearer picture of how parental education continues to influence decisions on demographic transitions and its impacts on intergenerational inequality.

## Appendix

**Table 5:** Complete results of logistic discrete-time analysis for first union

	Model A	Model B	Model C
	*b* (SE)	*b* (SE)	*b* (SE)
Father’s Education	–0.0032**	–0.0023**	–0.0002
	(0.0003)	(0.0004)	(0.0008)
Mother’s Education	–0.0049**	–0.0042**	–0.0035**
	(0.0005)	(0.0005)	(0.0011)
Respondent’s Education		–0.0048**	–0.0050**
		(0.0004)	(0.0004)
Interactions			
Father’s education × Cohort			–0.0000
			(0.0000)
Father’s education × Economic growth			–0.0002
			(0.0002)
Father’s education × Age			0.0005**
			(0.0001)
Father’s education × Female			–0.0028**
			(0.0007)
Mother’s education × Cohort			0.0000
			(0.0001)
Mother’s education × Economic growth			–0.0003
			(0.0003)
Mother’s education × Age			0.0005**
			(0.0001)
Mother’s education × Female			0.0005
			(0.0010)
Controls			
Age	0.1866**	0.1888**	0.1499**
	(0.0037)	(0.0037)	(0.0055)
Age^2^	–0.0469**	–0.0474**	–0.0475**
	(0.0009)	(0.0009)	(0.0009)
Age^3^	0.0020**	0.0020**	0.0020**
	(0.0001)	(0.0001)	(0.0001)
Cohort	–0.0158**	–0.0156**	–0.0137**
	(0.0015)	(0.0016)	(0.0023)
Cohort^2^	0.0014	0.0011	0.0015
	(0.0010)	(0.0010)	(0.0011)
Cohort^3^	0.0276**	0.0287**	0.0300**
	(0.0051)	(0.0051)	(0.0052)
Survey (ref. = OG 1998)			
NESTOR	–0.2386**	–0.3111**	–0.2977**
	(0.0666)	(0.0669)	(0.0670)
ESR telepanel	–0.5229**	–0.5661**	–0.5572**
	(0.0353)	(0.0355)	(0.0355)
OG 1993	0.0877**	0.0708**	0.0693**
	(0.0188)	(0.0189)	(0.0189)
OG 2003	0.0535**	0.0364*	0.0362*
	(0.0183)	(0.0184)	(0.0184)
OG 2008	0.1592**	0.1336**	0.1301**
	(0.0187)	(0.0189)	(0.0189)
FE 2003	–0.3297**	–0.3736**	–0.3728**
	(0.0386)	(0.0387)	(0.0388)
FE 2009	0.0250	–0.0553	–0.0629
	(0.0379)	(0.0380)	(0.0378)
Female (ref. = male)	0.4958**	0.4975**	0.5962**
	(0.0191)	(0.0191)	(0.0370)
Economic growth	0.0115**	0.0114**	0.0289**
	(0.0036)	(0.0036)	(0.0084)
Religion of father (ref. = no religion)			
Catholic	–0.1255**	–0.1206**	–0.1195**
	(0.0300)	(0.0298)	(0.0298)
Protestant	–0.1318**	–0.1275**	–0.1249**
	(0.0267)	(0.0267)	(0.0265)
Other religion	0.0058	–0.0081	–0.0061
	(0.0441)	(0.0440)	(0.0441)
Missing religion	–0.1474**	–0.1729**	–0.1724**
	(0.0450)	(0.0449)	(0.0448)
Religion of mother (ref. = no religion)			
Catholic	–0.0463	–0.0382	–0.0357
	(0.0306)	(0.0304)	(0.0304)
Protestant	–0.0537*	–0.0439	–0.0433
	(0.0273)	(0.0273)	(0.0273)
Other religion	–0.0517	–0.0596	–0.0660
	(0.0431)	(0.0430)	(0.0431)
Missing religion	–0.6490**	–0.6552**	–0.6581**
	(0.0582)	(0.0580)	(0.0580)
Divorced parents <18	0.1992**	0.1855**	0.1841**
	(0.0337)	(0.0336)	(0.0336)
Female × Age	–0.1534**	–0.1528**	–0.1528**
	(0.0052)	(0.0052)	(0.0052)
Female × Age^2^	0.0070**	0.0068**	0.0066**
	(0.0011)	(0.0011)	(0.0011)
Female × Age^3^	0.0001	0.0001	0.0001
	(0.0001)	(0.0001)	(0.0001)
Female × Cohort	–0.0017	–0.0005	0.0014
	(0.0020)	(0.0020)	(0.0020)
Female × Cohort^2^	–0.0029*	–0.0030*	–0.0026*
	(0.0013)	(0.0013)	(0.0013)
Female × Cohort^3^	0.0050	0.0036	0.0042
	(0.0060)	(0.0060)	(0.0060)
Constant	–3.9169**	–3.6743**	–3.7693**
	(0.0278)	(0.0336)	(0.0441)

**p* < .05; ***p* < .01

**Table 6:** Complete results of logistic discrete-time analysis for first marriage

	Model A	Model B	Model C
	*b* (SE)	*b* (SE)	*b* (SE)
Father’s Education	–0.0066^**^	–0.0051^**^	–0.0084^**^
	(0.0004)	(0.0005)	(0.0011)
Mother’s Education	–0.0077^**^	–0.0065^**^	–0.0146^**^
	(0.0006)	(0.0006)	(0.0016)
Respondent’s Education		–0.0072^**^	–0.0073^**^
		(0.0004)	(0.0004)
Interactions			
Father’s education × Cohort			0.0000
			(0.0000)
Father’s education × Economic growth			0.0006^**^
			(0.0002)
Father’s education × Age			0.0007^**^
			(0.0001)
Father’s education × Female			–0.0007
			(0.0009)
Father’s education × Cohabitation			0.0045^**^
			(0.0009)
Mother’s education × Cohort			–0.0002^**^
			(0.0001)
Mother’s education × Economic growth			0.0000
			(0.0003)
Mother’s education × Age			0.0010^**^
			(0.0001)
Mother’s education × Female			0.0031^*^
			(0.0013)
Mother’s education × Cohabitation			0.0102^**^
			(0.0012)
Controls			
Age	0.0651^**^	0.0676^**^	0.0097
	(0.0041)	(0.0041)	(0.0057)
Age^2^	–0.0399^**^	–0.0403^**^	–0.0412^**^
	(0.0008)	(0.0008)	(0.0008)
Age^3^	0.0021^**^	0.0021^**^	0.0021^**^
	(0.0001)	(0.0001)	(0.0001)
Cohort	–0.0672^**^	–0.0677^**^	–0.0610^**^
	(0.0019)	(0.0019)	(0.0030)
Cohort^2^	–0.0053^**^	–0.0056^**^	–0.0045^*^
	(0.0015)	(0.0015)	(0.0015)
Cohort^3^	0.0344^**^	0.0376^**^	0.0396^**^
	(0.0068)	(0.0068)	(0.0069)
Survey (ref. = OG 1998)			
NESTOR	–0.7634^**^	–0.8668^**^	–0.8741^**^
	(0.0826)	(0.0827)	(0.0833)
ESR telepanel	–1.0445^**^	–1.1120^**^	–1.1136^**^
	(0.0479)	(0.0481)	(0.0481)
OG 1993	0.0032	–0.0294	–0.0273
	(0.0211)	(0.0212)	(0.0212)
OG 2003	0.0654^**^	0.0311	0.0303
	(0.0206)	(0.0208)	(0.0208)
OG 2008	–0.3559^**^	–0.3986^**^	–0.4055^**^
	(0.0239)	(0.0242)	(0.0242)
FE 2003	–0.2439^**^	–0.3202^**^	–0.3205^**^
	(0.0393)	(0.0396)	(0.0397)
FE 2009	0.0359	–0.0125	–0.0192
	(0.0323)	(0.0325)	(0.0325)
Female (ref. = male)	0.2406^**^	0.2471^**^	0.1797^**^
	(0.0224)	(0.0222)	(0.0451)
Economic growth	0.0500^**^	0.0494^**^	0.0231^*^
	(0.0041)	(0.0041)	(0.0102)
Religion of father (ref. = no religion)			
Catholic	0.0675^*^	0.0771^*^	0.0756^**^
	(0.0299)	(0.0295)	(0.0296)
Protestant	0.1300^**^	0.1339^**^	0.1292^**^
	(0.0295)	(0.0295)	(0.0296)
Other religion	0.3885^**^	0.3811^**^	0.3657^**^
	(0.0439)	(0.0439)	(0.0439)
Missing religion	–0.0330	–0.0611	–0.0655
	(0.0486)	(0.0482)	(0.0483)
Religion of mother (ref. = no religion)			
Catholic	0.1085^**^	0.1257^**^	0.1203^**^
	(0.0309)	(0.0306)	(0.0306)
Protestant	0.1338^**^	0.0158^**^	0.1525^**^
	(0.0306)	(0.0306)	(0.0307)
Other religion	0.2448^**^	0.2272^**^	0.2154^**^
	(0.0433)	(0.0434)	(0.0435)
Missing religion	–0.1390^*^	–0.1547^**^	–0.1572^**^
	(0.0619)	(0.0612)	(0.0613)
Divorced parents <18	–0.1484^**^	–0.1829^**^	–0.1790^**^
	(0.0387)	(0.0386)	(0.0386)
Cohabitation	1.0113^**^	1.0147^**^	0.4794^**^
	(0.0159)	(0.0159)	(0.0412)
Female × Age	–0.1354^**^	–0.1346^**^	–0.1384^**^
	(0.0058)	(0.0058)	(0.0059)
Female × Age^2^	0.0115^**^	0.0113^**^	0.0111^**^
	(0.0009)	(0.0009)	(0.0009)
Female × Age^3^	–0.0002^*^	–0.0002^*^	–0.0002^*^
	(0.0001)	(0.0001)	(0.0001)
Female × Cohort	0.0011	0.0024	0.0009
	(0.0024)	(0.0024)	(0.0023)
Female × Cohort^2^	–0.0019	–0.0018	–0.0024
	(0.0018)	(0.0018)	(0.0019)
Female × Cohort^3^	0.0038	0.0028	0.0032
	(0.0082)	(0.0082)	(0.0083)
Constant	–4.9578^**^	–4.6083^**^	–4.1998^**^
	(0.0353)	(0.0398)	(0.0577)

**p* < .05; ***p* < .01

**Table 7:** Complete results of the multinomial analysis, with base category marriage

	Cohabitation vs. Marriage			Single vs. Marriage		
	Model A	Model B	Model C	Model A	Model B	Model C
	*b* (SE)	*b* (SE)	*b* (SE)	*b* (SE)	*b* (SE)	*b* (SE)
Father’s Education	0.0098**	0.0076**	0.0095**	0.0089**	0.0068**	0.0071**
	(0.0007)	(0.0007)	(0.0016)	(0.0005)	(0.0006)	(0.0012)
Mother’s Education	0.0111** (0.0010)	0.0095** (0.0010)	0.0109** (0.0023)	0.0127** (0.0008)	0.0112** (0.0008)	0.0124** (0.0019)
Respondent’s Education		0.0101**	0.0099**		0.0094**	0.0098**
		(0.0008)	(0.0008)		(0.0005)	(0.0005)
Interactions						
Father’s education × Cohort			–0.0002* (0.0001)			0.0002** (0.0001)
Father’s education × Economic growth			0.0002 (0.0004)			–0.0000 (0.0003)
Father’s education × Age			–0.0003 (0.0002)			–0.0006** (0.0002)
Father’s education × Female			–0.0000 (0.0016)			0.0020 (0.0012)
Mother’s education × Cohort			–0.0000			0.0003**
			(0.0001)			(0.0001)
Mother’s education × Economic growth			0.0004			0.0002
			(0.0006)			(0.0004)
Mother’s education × Age			–0.0009**			–0.0009**
			(0.0003)			(0.0002)
Mother’s education × Female			0.0006			–0.0002
			(0.0023)			(0.0018)
Controls						
Age	–0.0037	0.0068	0.0276*	–0.1912**	–0.1938**	–0.1464**
	(0.0074)	(0.0074)	(0.0114)	(0.0055)	(0.0055)	(0.0085)
Age^2^	0.0231**	0.0240**	0.0242**	0.0611**	0.0619**	0.0621**
	(0.0019)	(0.0019)	(0.0019)	(0.0016)	(0.0016)	(0.0016)
Age^3^	–0.0011**	–0.0012**	–0.0012**	–0.0027**	–0.0027**	–0.0027**
	(0.0001)	(0.0001)	(0.0001)	(0.0001)	(0.0001)	(0.0001)
Cohort	0.1112**	0.1108**	0.1211**	0.0857**	0.0852**	0.0663**
	(0.0038)	(0.0038)	(0.0055)	(0.0024)	(0.0024)	(0.0039)
Cohort^2^	–0.0201**	–0.0195**	–0.0190**	–0.0016	–0.0010	–0.0030
	(0.0025)	(0.0025)	(0.0026)	(0.0019)	(0.0019)	(0.0019)
Cohort^3^	–0.0182	–0.0198	–0.0157	–0.0697**	–0.0711**	–0.0743**
	(0.0150)	(0.0151)	(0.0150)	(0.0074)	(0.0075)	(0.0076)
Survey (ref. = OG 1998)						
NESTOR	0.5676*	0.7372**	0.8209*	0.2472**	0.4081**	0.3615**
	(0.2768)	(0.2768)	(0.2751)	(0.0728)	(0.0733)	(0.0735)
ESR telepanel	0.1489*	0.2501**	0.2558**	0.5797**	0.6754**	0.6636**
	(0.0716)	(0.0720)	(0.0720)	(0.0452)	(0.0456)	(0.0455)
OG 1993	0.0194	0.0603	0.0567	0.0809**	0.0421	0.0428
	(0.0377)	(0.0379)	(0.0380)	(0.0280)	(0.0281)	(0.0281)
OG 2003	–0.0082	0.0373	0.0388	–0.0436	0.0001	–0.0026
	(0.0367)	(0.0369)	(0.0369)	(0.0270)	(0.0271)	(0.0271)
OG 2008	–0.1319**	–0.0633	–0.0668	–0.2275**	–0.1614**	–0.1651**
	(0.0374)	(0.0378)	(0.0378)	(0.0278)	(0.0282)	(0.0282)
FE 2003	0.0970	0.2041*	0.2097**	0.3807**	0.4824**	0.4915**
	(0.0755)	(0.0758)	(0.0760)	(0.0500)	(0.0503)	(0.0506)
FE 2009	–0.1085	–0.0243	–0.0289	–0.0052	0.0762	0.0743
	(0.0713)	(0.0715)	(0.0711)	(0.0415)	(0.0418)	(0.0418)
Female (ref. = male)	–0.1830**	–0.1863**	–0.1975**	–0.6318**	–0.6348**	–0.7066**
	(0.0411)	(0.0411)	(0.0768)	(0.0316)	(0.0316)	(0.0600)
Economic growth	–0.0367**	–0.0366**	–0.0579**	–0.0198**	–0.0198**	–0.0245*
	(0.0075)	(0.0075)	(0.0182)	(0.0046)	(0.0046)	(0.0120)
Religion of father (ref. = no religion)						
Catholic	–0.2814**	–0.2947**	–0.2912**	–0.0495	–0.0622	–0.0650
	(0.0586)	(0.0582)	(0.0583)	(0.0420)	(0.0415)	(0.0414)
Protestant	–0.4254**	–0.4360**	–0.4288**	–0.1062**	–0.1161**	–0.1179**
	(0.0540)	(0.0539)	(0.0536)	(0.0383)	(0.0382)	(0.0380)
Other religion	–0.8488**	–0.8254**	–0.8480**	–0.4370**	–0.4155**	–0.4224**
	(0.0923)	(0.0923)	(0.0923)	(0.0558)	(0.0557)	(0.0554)
Missing religion	–0.2419*	–0.1908*	–0.1905*	–0.0172	–0.0303	–0.0304
	(0.0889)	(0.0888)	(0.0890)	(0.0669)	(0.0667)	(0.0665)
Religion of mother (ref. = no religion)						
Catholic	–0.2135**	–0.2273**	–0.2310**	–0.1062*	–0.1189**	–0.1204**
	(0.0601)	(0.0598)	(0.0598)	(0.0438)	(0.0433)	(0.0432)
Protestant	–0.3065**	–0.3273**	–0.3389**	–0.1553**	–0.1749**	–0.1768**
	(0.0557)	(0.0539)	(0.0553)	(0.0407)	(0.0406)	(0.0404)
Other religion	–0.6987**	–0.6871**	–0.6700**	–0.3495**	–0.3399**	–0.3152**
	(0.0898)	(0.0898)	(0.0899)	(0.0557)	(0.0558)	(0.0556)
Missing religion	–0.5567**	–0.5347**	–0.5525**	0.2998**	0.3211**	0.3225**
	(0.1150)	(0.1147)	(0.1150)	(0.0848)	(0.0844)	(0.0839)
Divorced parents <18	0.5441**	0.5752**	0.5714**	0.1986**	0.2280**	0.2272**
	(0.0706)	(0.0706)	(0.0706)	(0.0584)	(0.0583)	(0.0584)
Female × Age	0.0873**	0.0852**	0.0858**	0.2039**	0.2019**	0.0203**
	(0.0103)	(0.0103)	(0.0104)	(0.0077)	(0.0077)	(0.0077)
Female × Age^2^	–0.0091**	–0.0088**	–0.0088**	–0.0135**	–0.0132**	–0.0130**
	(0.0022)	(0.0022)	(0.0022)	(0.0019)	(0.0018)	(0.0018)
Female × Age^3^	0.0001	0.0001	0.0001	0.0001	0.0001	0.0001
	(0.0002)	(0.0002)	(0.0002)	(0.0001)	(0.0001)	(0.0001)
Female × Cohort	–0.0091	–0.0118*	–0.0120*	–0.0085**	–0.0110**	–0.0125**
	(0.0047)	(0.0047)	(0.0048)	(0.0030)	(0.0030)	(0.0030)
Female × Cohort^2^	0.0015	0.0016	0.0012	0.0080**	0.0081**	0.0077**
	(0.0033)	(0.0033)	(0.0033)	(0.0024)	(0.0025)	(0.0025)
Female × Cohort^3^	0.0533**	0.0566**	0.0574**	0.0192*	0.0224*	0.0214*
	(0.0183)	(0.0183)	(0.0184)	(0.0092)	(0.0092)	(0.0093)
Constant	0.4449**	–0.0463	–0.1614	4.8619**	4.4068**	4.3405**
	(0.0590)	(0.0709)	(0.0933)	(0.0464)	(0.0517)	(0.0700)

**p* < .05; ***p* < .01

**Table 8:** ISLED scores and their label (information from Schröder and Ganzeboom 2013)

Label	ISLED Score
Not Completed Primary School	16.55
Primary School or First Stage of Basic Education	22.98
Lower Secondary School, Technical Training [lbo]	29.34
Lower Secondary School, Theoretical Training [mulo,mavo]	45.27
Short Upper Secondary Professional Education [kmbo, vhbo]	45.70
Upper Secondary Professional Education [mbo]	52.70
Higher Secondary School [mms, havo]	62.30
Postecondary, Nontertiary Education [mbo plus]	64.58
Pre-Scientific Secondary School [hbs, vwo]	71.92
Tertiary Professional Education [hbo]	77.93
Tertiary Scientific Education, University	87.13
Tertiary Post-Scientific Education [teachers, doctors]	90.63
Second Stage of Tertiary Education, PhD Education	94.62

## Footnotes

1 White and Royston (2009) recommend using the Nelson-Aalen estimator in the imputation model.

2 This approach will underestimate some of the randomness in the process of educational attainment. However, given the highly stratified nature of the Dutch educational system, this assumption is reasonable.

3 Age is derived from information on year and month of birth. In 25.8 % of the cases, only information on year of birth was available. In these cases, month of birth was randomly imputed.

4 To facilitate interpretation and model convergence, we center age and cohort. For age, we center it on the mean age of entry into a partner relationship. In addition, we divide the quadratic and cubic terms for cohort by, respectively, 10 and 100.

5 One could argue that the effect of parental education on entry into marriage does not diminish because children are cohabiting but rather because they have left the parental home. Because information on leaving home is missing in part of the data sets, we checked this in a subsample (results available upon request). The effect of parental education is stronger when children are still living in the parental home than after they left home. However, the interaction between unmarried cohabitation and parental education remains significant as well.
